# The epidemic of loneliness

**DOI:** 10.1016/j.eclinm.2023.102395

**Published:** 2023-12-15

**Authors:** eClinicalMedicine

Although December is often associated with social connection and togetherness, in reality, this time of year can be among our most lonely. Christmas, Hanukkah, Kwanzaa, New Year, and Winter Solstice, among others, are typically times for strong social ties and coming together of families and friends. Unfortunately, for those experiencing social isolation, these events can place additional weight on feelings of loneliness, with dire consequences for both mental and physical health. Mind, a mental health charity, defines loneliness as “the feeling we have when our need for social contact and relationships isn’t met” and a personal experience that differs for everyone; it has also been defined as the discrepancy between someone’s desired and actual social relationships. It is important to distinguish social isolation and loneliness because, for some, being alone does not cause feelings of loneliness and others might experience loneliness when surrounded by people. A systematic review and meta-analysis assessing the prevalence of loneliness concluded that a substantial proportion of the population worldwide experience problematic levels of loneliness.

The list of both mental and physical health conditions that have been associated with loneliness and poor social connections is remarkably long—from impaired cognitive function, depression, anxiety, and increased risk of suicide, to cardiovascular disease, diabetes, and infectious diseases. Although the cause of these associations remains unconfirmed, it has been posited to be a result of emotional stress caused by being alone (ie, increased cortisol levels). Earlier in human evolution it was far more dangerous to be alone and community provided safety as well as shared resources, therefore, avoiding being alone had an evolutionary advantage and shapes our behaviour to this day. In a recent study in *eClinicalMedicine*, social isolation and loneliness were both associated with an elevated risk of type 2 diabetes, with Mendelian randomisation suggesting a possible causal association. Additionally, a report from the National Academies of Sciences, Engineering, and Medicine detailed some startling findings on loneliness and social isolation in older adults concluding that social isolation significantly increased an individual’s risk of premature death from all causes, rivalling the risks associated with smoking, obesity, and physical inactivity. Social isolation was associated with around a 50% increased risk of dementia, a 29% increased risk of heart disease, and a 32% increased risk of stroke. The report also highlighted higher rates of loneliness in the LGBTQ+ community and in migrants. Research has shown that loneliness can be associated with a range of factors, such as age, care responsibilities, employment status, gender, migration status, race, sexual orientation, and socioeconomic status.

In recent years, we have seen a greater awareness of the harms of loneliness and social isolation, especially following lockdowns as a result of the COVID-19 pandemic. The increase in remote working has also opened up discussions around the loss of social connectedness and the impact of isolation on mental health. Although this heightened awareness is cause for hope of positive change, public campaigns to address loneliness are predominantly aimed at older people. Evidence suggests that the relationship between social isolation and age is not clear cut. The current generation of younger people (born 1997–2012; Gen Z) has experienced multiple challenges for social interactions, with their education disrupted by the pandemic and the rise in social media use leading to less in-person communication. A recent study in the journal *International Psychogeriatrics* suggested we experience an increase in loneliness during three specific transitional periods in our lives: our late-20s, mid-50s, and late-80s. According to a survey published in 2020 by the health insurer Cigna, feelings of isolation were prevalent across generations, with those aged 18–22 years having the highest average loneliness score while those aged 55–73 years had the lowest, likely partly as a result of greater social media use in younger people. The report found loneliness to be more common among people with high levels of engagement with social media compared with those with low levels.

Given the substantial evidence base indicating the number of conditions associated with loneliness and social isolation, prevention strategies must be prioritised within clinical interactions as an integral part of health care. We are seeing changes in this direction. As more and more data are pouring in on the effects of loneliness on various health conditions and life expectancy, public health experts are getting behind projects to establish causes and work on solutions. Social prescribing, which “connects people to activities, groups, and services in their community” has been implemented by the UK National Health Service and has also been taken up in Ireland and the Netherlands. During the course of the UN Decade of Healthy Ageing (2021–30), activities will be addressing social isolation and loneliness as a theme that cuts across four main action areas. While this is welcome progress, there are far fewer initiatives for other susceptible groups, such as younger people and migrants, who are often left behind within the loneliness epidemic. Public health campaigns to reduce stigma and encourage social connection are a good start, but it is in our own communities that we can make the biggest difference. Communities and health-care systems must prioritise social connections across all generations as a vital pillar of overall wellbeing, not just during the festive season but all year round.

